# Recombinant GH3 β-glucosidase stimulated by xylose and tolerant to furfural and 5-hydroxymethylfurfural obtained from *Aspergillus nidulans*

**DOI:** 10.1186/s40643-024-00784-2

**Published:** 2024-07-29

**Authors:** Diandra de Andrades, Robson C. Alnoch, Gabriela S. Alves, Jose C. S. Salgado, Paula Z. Almeida, Gabriela Leila Berto, Fernando Segato, Richard J. Ward, Marcos S. Buckeridge, Maria de Lourdes T. M. Polizeli

**Affiliations:** 1 Department of Biology, Faculty of Philosophy, Sciences and Letters of Ribeirão Preto, University of São Paulo, Ribeirão Preto, SP 14040-901 Brazil; 2 Department of Biochemistry and Immunology, Faculty of Medicine of Ribeirão Preto, University of São Paulo, Ribeirão Preto, SP 14049-900 Brazil; 3 Laboratory of Enzymology and Molecular Biology of Microorganisms, Institute of Biology, Campinas State University (UNICAMP), Campinas, 13083-970 SP Brazil; 4 Department of Chemistry, Faculty of Philosophy, Sciences and Letters of Ribeirão Preto, University of São Paulo, Ribeirão Preto, SP 14040-901 Brazil; 5 Department of Biotechnology, Lorena School of Engineering, University of São Paulo, Lorena, 12602-810 Brazil; 6 Bioscience Institute, University of São Paulo, São Paulo, 05508-090 Brazil

**Keywords:** Enzymatic hydrolysis, Xylose-stimulated, Tolerant to biomass products, Homologous expression

## Abstract

The β-glucosidase gene from *Aspergillus nidulans* FGSC A4 was cloned and overexpressed in the *A. nidulans* A773. The resulting purified β-glucosidase, named AnGH3, is a monomeric enzyme with a molecular weight of approximately 80 kDa, as confirmed by SDS-PAGE. Circular dichroism further validated its unique canonical barrel fold (β/α), a feature also observed in the 3D homology model of AnGH3. The most striking aspect of this recombinant enzyme is its robustness, as it retained 100% activity after 24 h of incubation at 45 and 50 ºC and pH 6.0. Even at 55 °C, it maintained 72% of its enzymatic activity after 6 h of incubation at the same pH. The kinetic parameters V_max_, K_M_, and Kcat/K_M_ for *ρ*-nitrophenyl-β-D-glucopyranoside (*ρ*NPG) and cellobiose were also determined. Using *ρ*NPG, the enzyme demonstrated a V_max_ of 212 U mg ^− 1^, K_M_ of 0.0607 mmol L ^− 1^, and K_cat_/K_M_ of 4521 mmol L ^− 1^ s ^− 1^ when incubated at pH 6.0 and 65 °C. The K_M_, V_max_, and K_cat_/K_M_ using cellobiose were 2.7 mmol L ^− 1^, 57 U mg ^− 1^, and 27 mmol ^–1^ s ^− 1^, respectively. AnGH3 activity was significantly enhanced by xylose and ethanol at concentrations up to 1.5 mol L ^− 1^ and 25%, respectively. Even in challenging conditions, at 65 °C and pH 6.0, the enzyme maintained its activity, retaining 100% and 70% of its initial activity in the presence of 200 mmol L ^− 1^ furfural and 5-hydroxymethylfurfural (HMF), respectively. The potential of this enzyme was further demonstrated by its application in the saccharification of the forage grass *Panicum maximum*, where it led to a 48% increase in glucose release after 24 h. These unique characteristics, including high catalytic performance, good thermal stability in hydrolysis temperature, and tolerance to elevated concentrations of ethanol, D-xylose, furfural, and HMF, position this recombinant enzyme as a promising tool in the hydrolysis of lignocellulosic biomass as part of an efficient multi-enzyme cocktail, thereby opening new avenues in the field of biotechnology and enzymology.

**Supplementary Information:**

The online version contains supplementary material available at 10.1186/s40643-024-00784-2.

## Introduction

Lignocellulosic biomasses, abundant and renewable resources, have been identified as one of the most promising alternatives to meet increasing energy demands. Numerous studies have demonstrated the extensive potential of lignocellulosic biomass for the sustainable production of second-generation biofuels and various biomolecules and biomaterials with high-added value (McKendry 2002; Isikgor and Becer 2015; Mussatto et al. 2021; Alnoch et al. 2022). These biomasses primarily originate from plant cell walls and typically comprise 40–60% cellulose, 20–40% hemicellulose, and 10–25% aromatic hydrocarbon lignin (Zoghlami and Paës 2019; Srivastava et al. 2019).

Cellulose, a long-chain homopolymer comprising D-anhydroglucopyranose units covalently linked by β-(1,4) glycosidic bonds, exhibits a high degree of polymerization (DP) and molecular weight (Brigham 2018; Acharya et al. 2021). Due to its polysaccharide structure, cellulose contains numerous hydroxyl groups in the D-glucose units. It forms robust intra- and intermolecular hydrogen bond networks, resulting in a compact crystalline structure (highly ordered region) (Alves et al. 2018; Zoghlami and Paës 2019; Michelin et al. 2020; Etale et al. 2023). Moreover, partial cellulose chains are irregularly arranged, constituting the amorphous region of cellulose (the disordered region). Thus, although cellulose ranks among the most recalcitrant materials, it is also one of the most abundant biomaterials on Earth, harboring significant biotechnological potential (Sun et al. 2016; Alves et al. 2018; Zoghlami and Paës 2019; Michelin et al. 2020).

The complete enzymatic conversion of the cellulose into monomeric sugars requires synergistic interactions among the cellulolytic complex enzymes, including endoglucanases, cellobiohydrolases, and β-glucosidases. Endo-1,4-β-glucanases (EC 3.2.1.4) hydrolyze the β-1,4 glycosidic bonds randomly within the amorphous cellulose structure, releasing oligosaccharides of varying DPs. Cellobiohydrolases (CBH, exo-1,4-β-glucanases, EC 3.2.1.91 and 3.2.1.176) cleave the ends of cellulose chains (both reduced and non-reduced), releasing oligosaccharides, primarily cellobiose units (Bajpai 2018; Hildén and Mäkelä 2018; Srivastava et al. 2019).

The third enzyme group in the complex comprises β-glucosidases (β-D-glucopyranoside glucohydrolase; EC 3.2.1.21), crucial for hydrolyzing β-1,4-glycosidic bond in oligosaccharides, aryl-, and alkyl β-glucosides, as well as disaccharides, releasing the glucose monomer (Chang et al. 2018; Salgado et al. 2018). Other essential enzymes include lytic polysaccharide monooxygenases (LPMOs, AA9 to 11 and AA12 to 17, EC 1.14.99.54 and EC 1.14.99.56), which utilize a copper-dependent oxidative mechanism to break cellulose chains, and carbohydrate-specific oxidoreductases such as cellobiose dehydrogenase (CDH, AA3, EC 1.1.99.18) and cello-oligosaccharide dehydrogenase (AA7, EC 1.1.99.-), which donate electrons to LPMOs during the carbohydrate oxidation process (Freitas et al. 2021b; Alnoch et al. 2023).

The β-glucosidases have been extensively studied for their broad applications in food, feed, textile, and paper industries (de Andrades et al.2019a; Mishra et al. 2019). They are critical enzymes in biorefineries, facilitating the release of sugar monomers through enzymatic saccharification of cellulose. By hydrolyzing oligosaccharides and cellobiose (which are potent inhibitors of the activities of most cellobiohydrolases and endoglucanases), β-glucosidases play a crucial role (Singhania et al. 2011; Fusco et al. 2018; Huang et al. 2021). Moreover, the synergism between cellulolytic complex enzymes is essential for biomass degradation, as it can accelerate and increase hydrolysis yield (Agrawal et al. 2018; Alves et al. 2018).

Compounds released during pretreatment of lignocellulosic biomass, such as furfural or hydroxymethylfurfural (HMF), may inhibit these enzymes or disrupt their synergistic effects. Consequently, there is an increasing demand for biocatalysts with improved properties for industrial applications, such as increased stability at high temperatures and wide pH range and tolerance to toxic compounds resulting from the process (Wojtusik et al. 2017; Alves et al. 2018).

Filamentous fungi are widely employed as hosts for protein production in various biotechnological applications. The main advantage of using microbial systems lies in their rapid growth on cost-effective substrates, along with well-known genetics and physiology (Kück and Hoff 2010; Ward 2012; Daly et al. 2017; Yan et al. 2023). *Aspergillus nidulans* is among the foremost laboratory model systems for protein cell-factory since it has a protein synthesis machinery well that can produce and secrete amounts of proteins (Lubertozzi and Keasling 2009; Fleissner and Dersch 2010; Segato et al. 2012). For instance, several CAZymes have been successfully overexpressed in the last few years using a high expression pEXPYR vector integrated into *A. nidulans* A773 host (Segato et al. 2012; Ribeiro et al. 2014; Velasco et al. 2020; Liu et al. 2021; Gonçalves et al. 2023; Alnoch et al. 2023). In brief, the pEXPYR vector contains the glucoamylase promoter (*glaAp*) and secretion peptide (*glaAsp*) of *Aspergillus niger*, which enables the maltose-induced expression, high-yield secretion and accumulation of the recombinant enzyme in the extracellular medium (Segato et al. 2012).

Considering all these aspects, this work aimed to report the cloning, production, purification, kinetics, and biochemical characterization of a β-glucosidase (GH3) expressed in homologous host *A. nidulans* A773. For this, the thermal and pH stabilities, the specificity on different substrates, and its tolerance to compounds commonly found in the reaction medium for the hydrolysis of lignocellulosic residues and high added value compounds production, such as glucose, xylose, ethanol, furfural, and HMF were evaluated. Additionally, the potential application of AnGH3 in the hydrolysis of cellulosic biomass using forage grass *P. maximum* was also analyzed.

## Materials and methods

### Reagents and suppliers

The following substrates *ρ*-nitrophenyl-β-D-glucopyranoside (*ρ*NPG), *ρ*-nitrophenyl-β-D-galactopyranoside (*ρ*NPGal), *ρ*-nitrophenyl-α-D-glucopyranoside (α-*ρ*NPG), *ρ*-nitrophenyl-β-D-cellobioside (*ρ*NPCel), *ρ*-nitrophenyl-β-D-xylopyranoside (*ρ*NPX), cellobiose, salicin, carboxymethylcellulose (CMC) and reagents 3,5-dinitrosalicylic acid (DNS), furfural (99%) and 5-(hydroxymethyl)furfural (99%) were purchased from Sigma-Aldrich (St. Louis, USA). Precision Plus Protein Dual Color Standards and Bradford Protein assay were obtained from Bio-Rad Laboratories (Hercules, CA, USA). All other reagents used were of analytical grade.

### Cloning, transformation, and screening of recombinant transformants

*A. nidulans* strains FGSC A4 and A773 (*pyrG89; wA3; pyroA4*) were obtained from the Fungal Genetics collection (FGSC, Kansas City, MO, USA). Genomic DNA extraction from *A. nidulans* FGSC A4 was carried out using the Wizard Genomic DNA Purification Kit (Promega, Madison, WI, USA). The oligonucleotides AnGH3F (forward, 5’-CATTACACCTCAGCAATGCGCTCTCTGATAAGATCCGGCG-3’; and AnGH3R (reverse, 5’-GTCCCGTGCCGGTTACTAGACGGTAAAGCTTCCCGTCAACCG-3’) were designed based on the GH3 coding sequence *angh3* (access number XM_655340.1) and amplified from genomic DNA by PCR and then cloned into the pEXPYR expression vector using the Gibson Assembly method (Gibson et al. 2009). The resulting construct, pEXPYR-*angh3*, was transformed into the *A*. *nidulans A773*. Minimal media without uracil and uridine was used to select positive clones (Segato et al. 2012). SDS-PAGE confirmed the expression of AnGH3 by recombinant strains.

### Expression and purification of recombinant AnGH3

The culture medium consisted of 10 g of glucose, pyridoxine (1 mg L^− 1^), 50 mL of Clutterbuck salts (30.4 g KH_2_PO_4_, 120 g NaNO_3_, 10.4 g MgSO_4_·7H_2_O and 10.4 g KCl, per liter), 1 mL of trace elements (5 g Na_2_EDTA, 2.2 g ZnSO_4_·7H_2_O, 0.11 g Na_2_MoO_4_·4H_2_O, 0.5 g MnCl_2_·4H_2_O, 0.5 g FeSO_4_·7H_2_O, 0.16 g CuSO_4_·5H_2_O, 0.16 g CoCl_2_·5H_2_O, and 1.1 g H_3_BO_3_ in 100 ml), pH 6.5 (Segato et al. 2012).

The crude extract obtained was filtered through a Büchner funnel with Whatman nº1 filter paper, concentrated by ultrafiltration, and equilibrated using a 10 kDa cut-off membrane (Hollow Fiber Cartridge GE Healthcare) coupled in QuixStand Benchtop Systems. Subsequently, the purification process was performed using an ÄKTA Purifier system (GE Healthcare). The crude extract was loaded into an anion exchange Hiprep Q FF column (GE Healthcare) previously equilibrated in 50 mmol L^− 1^ phosphate buffer, pH 6.5, with a flow rate of 1 mL min^− 1^. Protein levels were monitored at 280 nm and eluted with a linear gradient from 0 to 1 mol L^− 1^ of NaCl (Alnoch et al. 2023). The recovered sample was then applied to a size exclusion chromatography (Superdex 75 10/300 GL GE Healthcare) column equilibrated in 50 mmol L^− 1^ phosphate buffer and 150 mmol L^− 1^ NaCl, pH 6.5, at a flow rate of 0.5 mL min^− 1^. Protein fractions exhibiting the highest β-glucosidase activities were selected via SDS-PAGE electrophoresis, pooled, ultrafiltered, and dialyzed using a 10 kDa cut-off membrane.

### Enzymatic assays

The β-glucosidase activity was quantified using *ρ*NPG or cellobiose as substrate. The *ρ*NPG assays utilized 50 µL of *ρ*NPG 4 mmol L^− 1^, 30 µL of phosphate buffer 100 mmol L^− 1^ at pH 6.0, and 20 µL of enzyme solution (4 µg). The assay was carried out at 65 °C for 2 min, stopped with 100 µL of 0.5 mol L^− 1^ Na_2_CO_3_ (pH 10). The released *ρ*–nitrophenolate was quantified at 410 nm using *ρ*–nitrophenol as a standard in the calibration curve.

Activity assays with cellobiose were performed using 50 µL of 25 mmol L^− 1^ cellobiose, 30 µL of 100 mmol L^− 1^ phosphate buffer at pH 6.0, and 20 µL of enzyme solution. The assay was carried out at 65 °C for 5 min, stopped by boiling for 5 min, followed by cooling in an ice bath. The amount of released glucose was measured using a glucose oxidase enzymatic assay kit (glucose liquiform, Labtest, Brazil). A volume of 10 µl of the previous mixture assay and 1 mL of the reagent solution kit were incubated at 37 °C for 10 min, and the absorbance was measured at 505 nm, with glucose used as standard in the calibration curve.

For both assays, one unit of enzyme activity (U) was defined as the amount of enzyme that catalyzes the release of 1 µmol of *ρ*-nitrophenolate or glucose per minute under assay conditions.

### Protein determination and electrophoresis analysis

The Bradford method was used to determine protein content (Bradford 1976), with bovine serum albumin as the standard. Electrophoresis of the protein samples (12% SDS − PAGE) was performed according to Laemmli (Laemmli 1970), using a molecular standard of 10 to 250 kDa (Precision Plus Protein™ Standards Bio − Rad). The gel was stained with Coomassie brilliant blue 0.05% (m v^–1^).

### Circular dichroism analysis

Circular dichroism analysis (CD) was performed in a Jasco-810 spectropolarimeter (JASCO Inc., Tokyo, Japan), as previously described (Alnoch et al. 2023). Protein samples (0.1 − 1 mg mL^− 1^) were mixed in 10 mmol L^− 1^ Tris − HCl buffer pH 7.0 and added in a quartz cuvette of 200 µL, with an optical path length of 0.1 mm. Data were collected at a scanning speed of 50 nm min^− 1^, a spectral bandwidth of 3 nm, and a response time of 1 s. Blank spectra with the buffer only were subtracted in all experiments. Measurements comprised six accumulations within the UV range UV of 190–250 nm. Analyses of the CD spectral data were perfomed with the DichroWeb server (Miles et al. 2022).

### Matrix-assisted laser desorption ionization-time of flight mass spectrometry (MALDI-TOF MS) analysis

For this procedure, 100 µg of purified AnGH3 (lyophilized) was resuspended in a solution containing 8.0 mol L^− 1^ urea (CH_4_N_2_O), 100 mmol L^− 1^ Tris − HCl, pH 8.5. Subsequently, the samples were treated with 100 µg of dithiothreitol (DTT) at 37 °C for 60 min, followed by alkylation with 300 µg of iodoacetamide at 37 °C for 60 min before tryptic digestion at 37 °C overnight. The mass spectra were obtained using a MALDI − TOF − TOF/MS (AutoFlex Max, Bruker). The obtained mass profiles were compared with peptide masses predicted through in silico digestion using the MS-Digest tool in Protein Prospector (Perkins et al. 1999) and MASCOT server (Chalkley et al. 2005).

### Sequence analysis and structural homology modeling

Sequence alignment was carried out using MEGA software version 11.0, employing the ClustalW algorithm (Thompson et al. 1994). Protein sequences were retrieved from the Protein Data Bank (PDB) (Berman et al. 2000). The ENDscript server (Robert and Gouet 2014) was utilized to predict the secondary structure and features of the amino acid sequence based on PDB templates. Homology modeling of AnGH3 was performed using AlphaFold2 (Jumper et al. 2021), integrated into UCSF ChimeraX (Pettersen et al. 2021).

### Deglycosylation analysis

The deglycosylation (*N* − glycosylation) assay was carried out using Endoglycosidase H (EndoH) (Roche, Mannheim, DE). For this assay, 5 µg of purified AnGH3 was mixed with 250 mU of EndoH in a sodium acetate buffer (50 mmol L^− 1^, pH 5.5) and maintained at 37 °C for 16 h. The samples were then analyzed using SDS − PAGE, and the samples were compared before and after treatment. To estimate the carbohydrate content, we compared the migration difference between the treated and untreated samples with that of the molecular mass standard (Wilson et al. 2009; Alnoch et al. 2023).

### Biochemical characterization of the AnGH3

#### Effect of temperature and pH on AnGH3 activity and stability

The effect of temperature on AnGH3 activity was assessed by measuring the hydrolysis of cellobiose across a temperature range of 35 to 85 °C, utilizing 50 mmol L^− 1^ sodium phosphate buffer at pH 6.0. AnGH3 was incubated for 24 h at 45 to 60 °C to evaluate the thermal stability.

The influence of pH on AnGH3 activity was investigated at 50 °C, over the range of 3.0 to 9.0, using: pH 3.0 − 5.5 (50 mmol L^− 1^ sodium citrate buffer), pH 5.5 − 7.5 (50 mmol L^− 1^ sodium phosphate buffer), and pH 7.5 − 9.0 (50 mmol L^− 1^ Tris − HCl buffer). The enzyme was incubated for 24 h at 25 °C across a pH range of 3.0 to 9.0 for pH stability determination. Residual activity was calculated relative to the initial enzyme activity.

#### Substrate specificity

To determine the substrate specificity of the enzyme, *ρ*NPG, *ρ*NPGal, *ρ*NPX, α-*ρ*NPG, *ρ*NPCel, CMC, cellobiose, lactose, and salicin, were utilized as substrates to evaluate the enzymatic activity in 100 mmol L^− 1^ phosphate buffer, pH 6.5, at 65 °C. For *ρ*NP substrates, the reactions were monitored by the *ρ*NP–releasing assay described above. Glucose release from cellobiose, lactose, and salicin was determined using the glucose oxidase-based kit, while reducing sugar release from other substrates was determined using the DNS method (Miller 1959).

#### Effect of different compounds on AnGH3 activity

The impact of various concentrations of xylose (0–2 mol L^− 1^), glucose (0–1 mol L^− 1^), furfural (0–200 mmol L^− 1^), 5–HMF (0–200 mmol L^− 1^), and ethanol (1–50% v/v), on AnGH3 activity were assessed by incubating the purified enzyme as described in Sect. 2.4. The residual activity was determined by comparing enzyme activity at each addictive concentration to the control without additives.

### Determination of kinetic parameters

The Michaelis-Menten constant (K_M_) and maximum velocity (V_max_) of AnGH3 (0.2 mg mL^− 1^) were determined using *ρ*NPG and cellobiose as substrates, with concentrations varying from 0.004 to 4 mmol L^− 1^ and 2 to 20 mmol L^− 1^, respectively. Assays were conducted at pH 6.0 and 65 °C, and the parameters were calculated using the Hanes method (Hanes 1932) with GraphPad Prism 8.0 software (GraphPad Software, LLC). Turnover number (k_cat_) and catalytic efficiency (K_cat_/K_M_) were also calculated.

### Enzymatic saccharification of biomass

To assess the potential application of AnGH3 in the hydrolysis of cellulosic biomass, the conversion of tropical forage grass (*Panicum maximum*) using the commercial preparation Celluclast^®^ 1.5 L (with or without AnGH3) was performed. The enzymatic conversion process was carried out using a substrate concentration of 3% (w/v, dry basis) in 50 mmol L^− 1^ citrate buffer pH 5.0. The commercial Cellulast^®^ 1.5 L (Novozymes, Denmark) load was 20 filter paper units (FPU) per gram of biomass, while the purified enzyme was loaded at 69 U per gram of biomass. The assay mixture was incubated in a Thermomixer C (Eppendorf, Hamburg, Germany) for 24 h at 50 °C and 1300 rpm. Following hydrolysis, the total glucose released was quantified using the glucose oxidase method (Labtest, Brazil), with glucose as the standard (de Andrades et al2019b). A control experiment was performed by hydrolysis of biomass without adding the enzyme to the reaction medium.

## Results and discussion

### Sequence analysis and molecular modeling

**Fig. 1:**
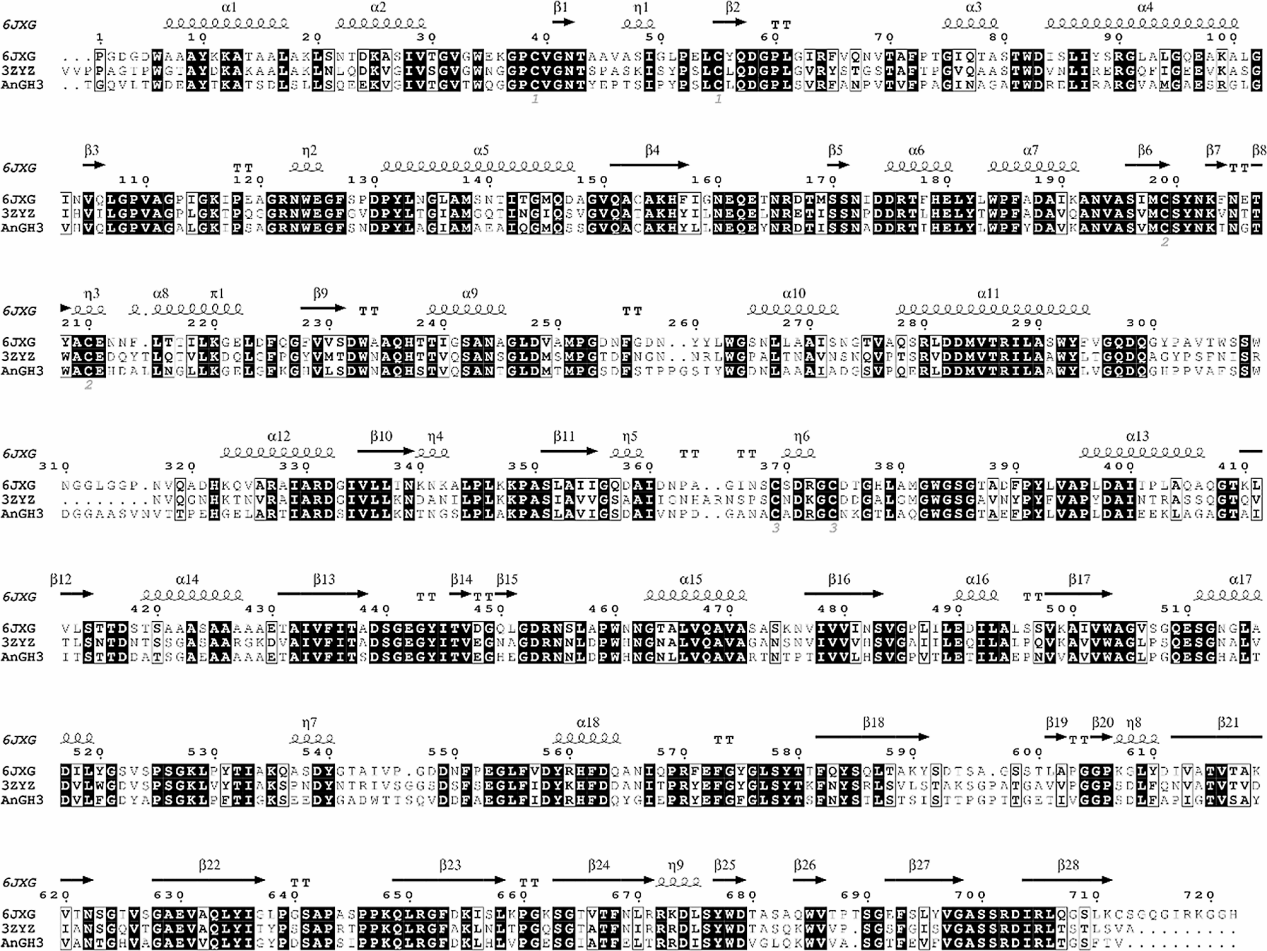
Multiple sequence alignment and secondary structure prediction of AnGH3 and PDB template. Access codes: *C. raphigera* [PDB: 6JXG] and *H. jecorina* [PDB: 3ZYZ]. Dark- and light-shaded boxes indicate conserved residues. Secondary structures representations: arrows represent β-strands, α-helices are represented by α, and β-turns are marked with TT. Sequence alignments were performed using Clustal (Thompson et al. 1994), and the figure was prepared using ESPriptS.

**Fig. 2:**
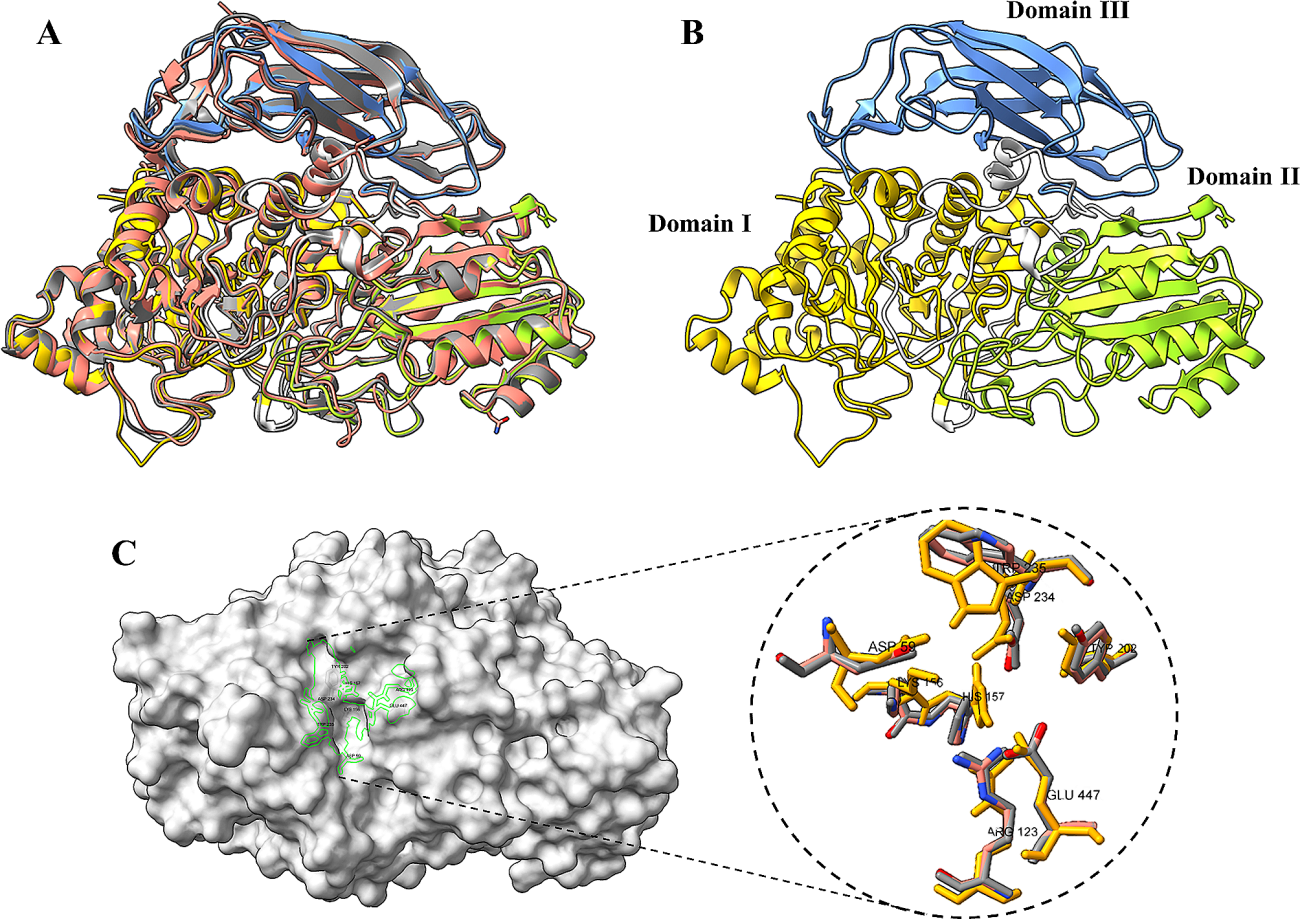
Schematic representation of the overall structure of AnGH3. **(A)** 3D model of AnGH3 superimposed with template structures from *C. raphigera* [PDB: 6JXG] (gray) and *H. jecorina* [PDB: 3ZYZ] (magenta). **(B)** 3D model of AnGH3 with the three domains are colored yellow (domain I), green (domain II), and blue (domain III). The two domain linker regions are shown in white. **(C)** Zoom-in of the modeled catalytic tunnel. The Figure was prepared using UCSF ChimeraX

The β-glucosidase AnGH3 from *A. nidulans* comprises 718 amino acid residues, including a 19 residues signal peptide, with the N-terminus in mature protein initiating with residues TGQVL (Fig. 1). To elucidate the characteristics of AnGH3 concerning the GH3 β-glucosidase utilized as a template for the 3D model, multiple sequence-structure alignments were conducted between the β-glucosidases from *Chaetomella raphigera* [PDB: 6JXG], and *Hypocrea jecorina* [PDB: 3ZYZ] (Fig. 2A). The 3D model and structure alignment revealed the distinctive structure of the β-glucosidase GH3, featuring a three domains structure, within domain I between the residues 1 to 312; domain II presenting a structural characteristic of α/β sandwich between residues 325 to 526 and the domain III composed of residues 578 to 718 with an immunoglobulin-like topology (Fig. 2B).

**Fig. 3:**
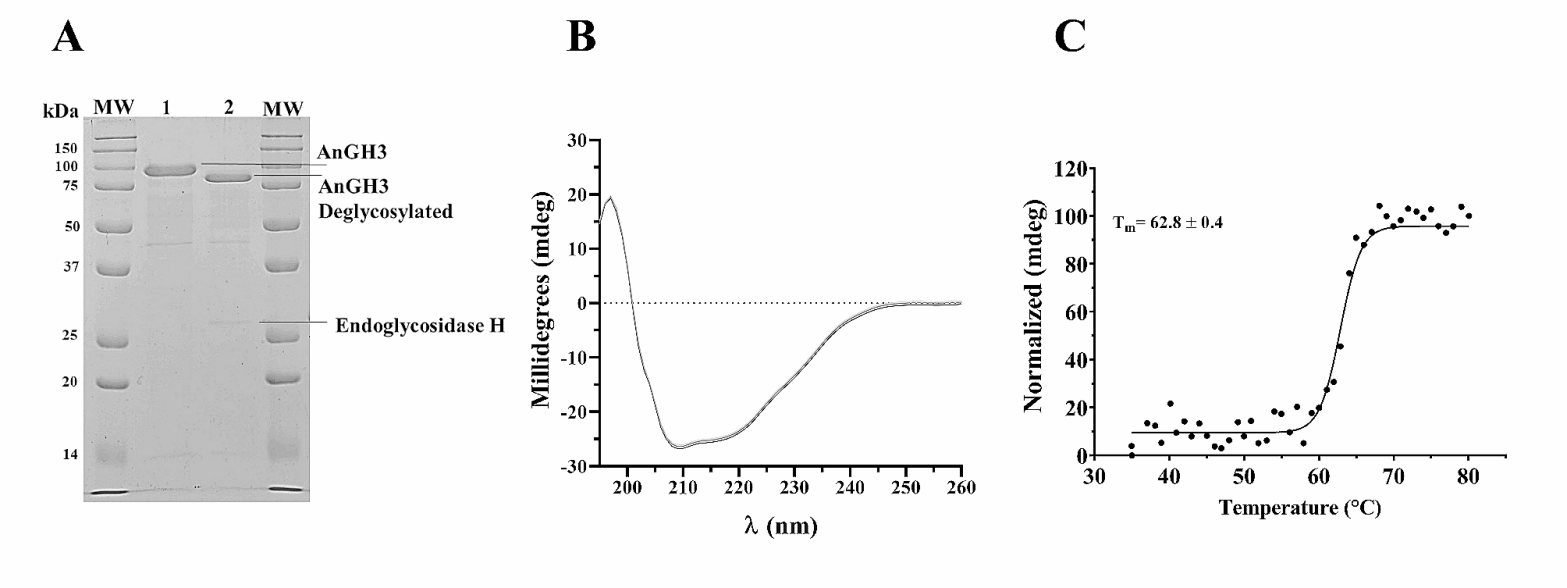
**(A)** Purification of the recombinant β-glucosidase AnGH3 from *A. nidulans*. SDS-PAGE of AnGH3 (line 1) from size exclusion Superdex 75 10/300 GL column and (lane 2) analysis of the enzymatic deglycosylation assay after digestion treatment with endoglycosidase H. MW: molecular weight standard ladder. **(B)** The secondary structure profile of purified AnGH3 was determined by CD spectroscopy. **(C)**. Thermal stability curve of AnGH3 determination of melting temperature (Tm)

Alignment of the structure and 3D model facilitated the identification of essential amino acid residues within the active site of AnGH3, aligning with analogous in other GH3 β-glucosidase (Karkehabadi et al. 2014; Kao et al. 2019). Asp^234^ and Glu^447^ serve as nucleophilic and acid/base residues, corresponding to Asp^232^ and Glu^442^ in the model PDB: 6JXG and Asp^236^ and Glu^441^ in the model PDB: 3ZYZ, indicating their consistent catalytic site function (Fig. 2C). Residues Asp^59^, Arg^123^, Lys^156^, His^157^_,_ and Trp^235^ constitute a substrate binding subsite, mirroring the crystal structures of *C. raphigera* and *H. jecorina* β-glucosidases in the same positions. However, Trp^235^ exhibits a distinct orientation compared to Trp^233^ in the structure PDB: 6JXG and Trp^237^ in PDB: 3ZYZ (Fig. 2C). The observed molecular weight variation (77.6 to 80.2 kDa) suggests glycosylation sites in AnGH3 (Fig. 3A). Three N–glycosylation sites (N^206^, N^321^ and N^346^) were predicted within the AnGH3 sequence using NetNGlyc – 1.0 Server (Gupta and Brunak 2002).

### Purification and identification AnGH3

Recombinant AnGH3 was successfully expressed in *A. nidulans* strain A773 and purified. AnGH3 is a monomeric enzyme, and the homogeneity of the purified AnGH3 was evidenced as a single band in SDS–PAGE (Fig. 3A), exhibiting an apparent molecular weight of approximately 80.2 kDa. Mass spectrometry confirmed that the band in the SDS-PAGE corresponds to the recombinant AnGH3 (Fig. S1).

Endo H was used for enzymatic deglycosylation assay. The profiles of native AnGH3 (80.2 kDa) and deglycosylated AnGH3 can be distinguished in lanes 1–2 (Fig. 3A). Deglycosylated AnGH3 exhibited an estimated molecular weight of 77.6 kDa, consistent with the theoretical molecular weight of 75.9 kDa for the AnGH3 sequence. Consequently, the results suggest a carbohydrate content of approximately 4% of the molecular weight of native AnGH3.

Circular dichroism spectra of AnGH3 revealed a negative band at 208 and 222 nm, characteristic of α–helix structure, and an upbeat band at 218 nm corresponding to β–sheets (Fig. 3B), matching content of α–helix, β–sheets and a random coil of 38%, 26% and 37%, respectively. These features align with the canonical barrel fold (β/α), as also observed in the 3D homology model of AnGH3. Figure 3C illustrates that the melting temperature (Tm) of the enzyme was 62.8 °C at pH 7.0. Similar results regarding the impact of temperature on secondary structure were reported for other GH3 enzymes (Méndez-Líter et al. 2017; Lima et al. 2020).

The specific activity of AnGH3 was determined to be 282 ± 17 U mg^− 1^, utilizing *ρ*NPG as substrate, at pH 6.0, 65 °C. This preparation was used for biochemical characterization assays.

### Biochemical characterization assays

#### Effect of pH and temperature on activity and stability of AnGH3

**Fig. 4:**
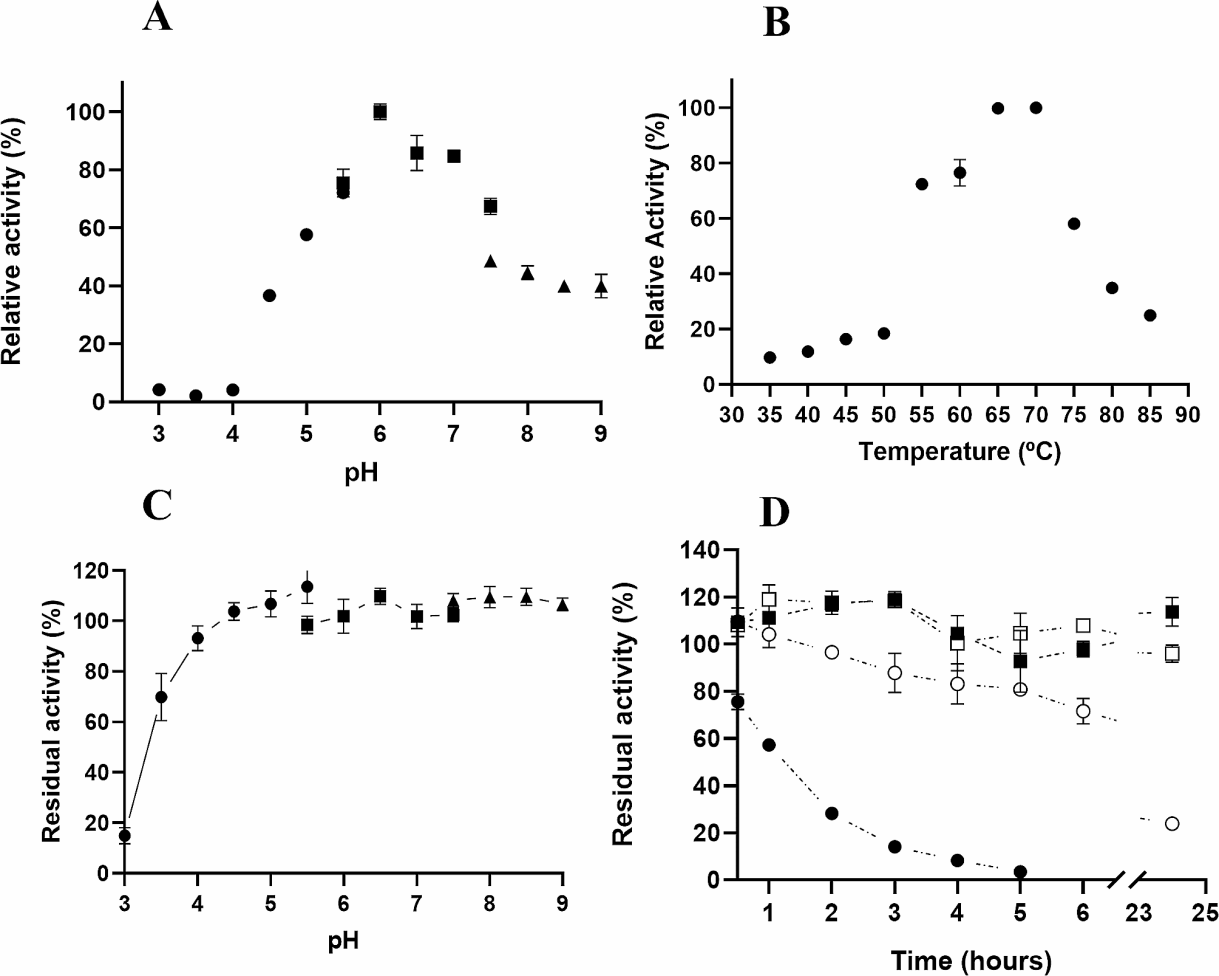
Biochemical characterization of AnGH3. **(A)** Influence of optimum pH on enzymatic activity. Residual activities were assayed at 65 °C in the pH range of 3.0-5.5 with sodium citrate (●), pH 5.5–7.5 with sodium phosphate (◼), and pH 7.5-9.0 with Tris − HCl buffers (▲). **(B)** Temperature influence on AnGH3. The purified enzyme was assayed at pH 6 in the 35–85 °C temperature range. **(C)** AnGH3 pH stability. The enzyme was incubated for 24 h, at 25 °C, at a pH range of 3.0 to 9.0. The residual activities were assayed at 65 °C and pH 6. The activity of the enzyme incubated in water was considered 100%. **(D)** Thermal denaturation of the purified AnGH3 at 45 °C (◼), 50 °C (□), 55 ºC (○) and 60 °C (●) up to 24 h. The residual activities concerning the initial enzyme activities were calculated. The residual activities were assayed at 65 °C and pH 6. The enzyme activity at incubation time zero was considered 100%

The effects of pH and temperature on the activity and stability of the AnGH3 were also analyzed. Figure 4A demonstrates that AnGH3 exhibits activity over a broad range of pH values (4.5–9.0), with a maximum at pH 6.0 (55.4 U mg^− 1^). At pH 7.5, the activity decreased to 67% or even to 48%, depending on the used buffer, indicating a significant influence of the buffer on AnGH3 activity, with sodium phosphate buffer providing more effective than Tris − HCl (Fig. 4A). AnGH3 remained fully active across a wide pH range (4.5 to 9.0), showing 100% of residual activity after 24 h of incubation (Fig. 4C). Additionally, AnGH3 retained 70 and 93% of its activity after 24 h of incubation at pH 3.5 and 4.0, respectively (Fig. 4C). These optimal pH conditions align with those described for recombinant enzymes such as BgL1 from *A. niger* BE-2 (Ali et al. 2016) and TrBgl2 from *Trichoderma reesei* (Solhtalab et al. 2019), confirming the reported optimal pH values between 4.0 and 6.0 for fungal β-glucosidases (Bonfá et al. 2018).

The optimal temperature for maximal AnGH3 activity was achieved around 65 and 70 °C, at pH 6.0 (56 U mg ^− 1^) (Fig. 4B). The soluble enzyme remained completely stable at 45 and 50 ºC after 24 h of the incubation (Fig. 4D). However, after 6 h of incubation at 55 °C, its enzymatic activity was reduced to 72%. Similar properties have been reported for other recombinant β-glucosidases expressed in *Pichia pastoris.* For instance, MtBgl3b from *Myceliophthora thermophila* exhibited maximum activity at 60 °C and pH 5.0, retaining about 90% of its relative activity at 60 °C for 120 min (Zhao et al. 2015). A BGL2 from *Neurospora crassa* displayed its highest activity at pH 5.4 and 60 °C, retaining 88.1 and 62.6% of its relative activity at 50 and 55 °C, respectively, after 20 min (Pei et al. 2016). The Bgl4 from *Penicillium funiculosum* NCL1 showed optimal activity at pH 5.0 and 60 °C, retaining 77% of its initial activity after 1 h of incubation at 60 °C (Ramani et al. 2015). These thermostable enzymes offer advantages in processes at higher temperatures, mainly concerning better substrate solubility and mass transfer (Turner et al. 2007; Sharma et al. 2019). Aiming to broaden the use of β-glucosidases in industry, it would be beneficial to use enzymes that tolerate non-mild conditions such as high temperatures and extreme pH values (Ouyang et al. 2023).

#### Substrate specificity of AnGH3

Various substrates were employed to explore the substrate specificity of AnGH3, and the findings are summarized in Table 1 and Fig. S2. The soluble enzyme demonstrated efficient hydrolysis of substrates featuring (1→4)-beta-glycosidic linkages, such as *ρ*NPG, cellobiose, *ρ*NPCel, and salicin. The most pronounced hydrolytic activity was observed with *ρ*NPG (282 U mg^− 1^), followed by cellobiose disaccharide (56 U mg^− 1^), the glycoside of salicin (40 U mg^− 1^), and *ρ*NPCel (4.5 U mg^− 1^). Additionally, the enzyme exhibited hydrolytic capability towards the glycoside of *ρ*NPX but showed no activity against CMC, *ρ*NPGal, and α-glycosidic bonds like α–*ρ*NPG.

**Table 1:** Substrate specificity of AnGH3

Substrate	Concentration	Specific activity (U mg^− 1^)
*ρ*NPG	2 mmol L^− 1^	282 ± 17
*ρ*NPGal	2 mmol L^− 1^	ND
*ρ*NPX	2 mmol L^− 1^	0.1 ± 0.01
α–*ρ*NPG	2 mmol L^− 1^	ND
*ρ*NPCel	2 mmol L^− 1^	4.5 ± 0.1
Cellobiose	1%	56 ± 0.3
CMC	1%	ND
Lactose	1%	ND
Salicin	1%	40 ± 0.8

ND, not detected

Several GH3 β-glucosidases capable of hydrolyzing not only *ρ*NPG and cellobiose but also other cello-oligosaccharides have been reported (Liu et al. 2012; Yan et al. 2012; Zhao et al. 2015; Ramani et al. 2015; Pei et al. 2016; Volkov et al. 2020; Dadwal et al. 2023). These enzymes display significant activity on diverse substrates and are often classified as broad-specificity β-glucosidases (Molina et al. 2016). The AnGH3 exhibited higher specificity activity for both *ρ*NPG and cellobiose compared to most family GH3 β-glucosidases, including MtBgl3b from *M. thermophila* (258 and 62 U mg^− 1^) (Zhao et al. 2015), nBgl3 from *A. fumigatus* (101 and 59 U mg^− 1^) (Liu et al. 2012), PtBglu3 from *Paecilomyces thermophila* (228 and 113 U mg^− 1^) (Yan et al. 2012), rAnBGL from *Penicillium verruculosum* (100 and 124 U mg^− 1^) (Volkov et al. 2020), MtBgl3c from *M. thermophila* (66 and 46 U mg^− 1^) (Dadwal et al. 2023), BGL2 from *N. crassa* (143 and 74 U mg^− 1^) (Pei et al. 2016).

According to Rajoka and colleagues (Rajoka et al. 2015), the variations in substrate specificity between *ρ*NPG and cellobiose arise from distinct interactions between various side chain residues of the β-glucosidase and each substrate. The authors performed structural analysis and docking studies with the thermostable β-glucosidase from *Thermotoga maritima* using cellobiose and *ρ*NP–linked substrates. In the enzyme-*ρ*NPG complex, the interaction occurred via three hydrogen bonds with the active site residues Glu^166^, Tyr^295^, and Asn^223^. In contrast, in the enzyme-cellobiose complex, the reaction involved residues Asn^223^, Ser^229^, and His^298^, forming hydrogen bonds with the ligand. Further structural investigations into these kinetic differences, particularly in fungi, are essential for future rational designs of β-glucosidases variants with improved properties.

#### Effect of biomass-derived compounds on AnGH3 activity

**Fig. 5:**
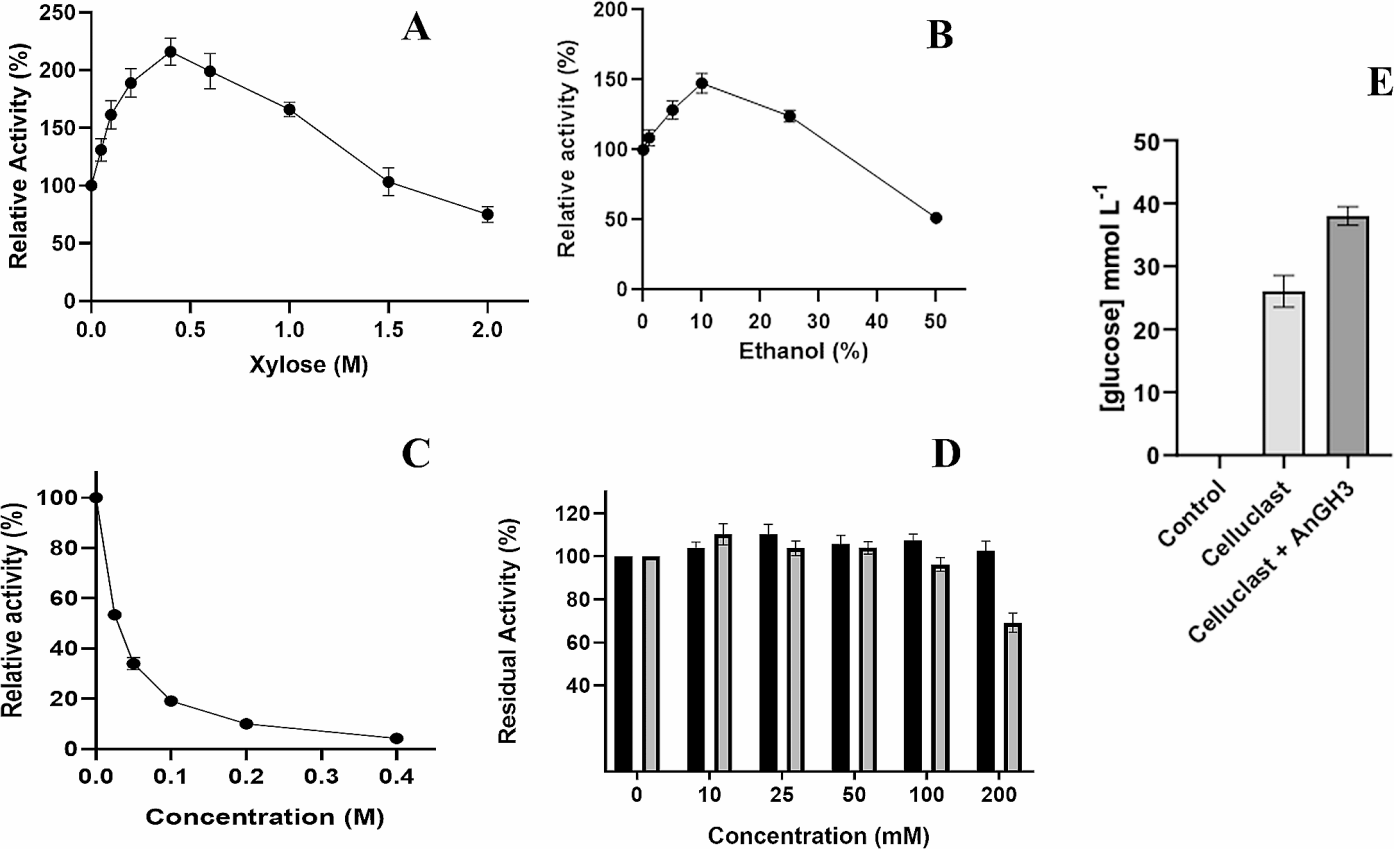
Effect of different compounds on AnGH3 activity. **(A)** Xylose effect on the AnGH3 activity. **(B)** Ethanol effect on the AnGH3 activity. **(C)** Effect of glucose concentration in AnGH3 activity. **(D)** Furfural (black bar) and 5–HMF (gray bar) effect on AnGH3 activity. The purified enzyme was assayed at 65 °C pH 6; the activity without additive was considered 100%. The residual activities concerning the initial enzyme activities were calculated. **(E)** Cellulosic biomass hydrolysis for 24 h at 50 °C, pH 5.0 using AnGH3 and Cellulast^®^ 1.5 L. All measurements were done in triplicates. Error bars show SD

Various concentrations of xylose (0–2 mol L^− 1^) (Fig. 5A), ethanol (0–50% v/v) (Fig. 5B**)**, glucose (0–1 mol L^− 1^) (Fig. 5C), furfural (0–200 mmol L^− 1^) and 5–HMF (0–200 mmol L^− 1^) (Fig. 5D) exhibited contrasting effects on AnGH3 activity. Surprisingly, AnGH3 activity was significantly stimulated by xylose (Fig. 5A), reaching a maximal 2.2-fold stimulation at 0.4 mol L^− 1^. Furthermore, the enzyme retained 100% activity even at high xylose concentrations of 1.5 mol L^− 1^. In contrast, in the presence of 25 mmol L^− 1^ and 200 mmol L^− 1^ of glucose (Fig. 5C), the relative enzymatic activity decreased by 53 and 10% relative to the control, respectively. Similar results were reported for β-glucosidase from the thermophilic fungus *Humicola brevis* var. *thermoidea*. The enzyme showed a maximal increase of about 1.7-fold at 200 mmol L^− 1^ xylose and retained around 30% of its activity in the presence of 30 mmol L^− 1^ glucose (Masui et al. 2012). However, xylose-stimulated β-glucosidases are usually reported with concomitant glucose stimulation, as observed in the intracellular β-glucosidases of the thermophilic fungi *H. insolens* (Souza et al. 2010), H. *grisea* var. *thermoidea* (Nascimento et al. 2010), *Scytalidium thermophilum* (Zanoelo et al. 2004), and the thermophilic bacterium *Anoxybacillus flavithermus* subsp. *yunnanensis* E13T (Liu et al. 2017). For these enzymes, studies suggest a regulatory binding site for glucose that is different from the active site, likely inducing conformational changes that stimulate the hydrolysis activity (Souza et al. 2013). GH3 β-glucosidases are uncommonly stimulated by glucose (and consequently xylose); on the other hand, in GH1 β-glucosidases, the glucose and xylose stimulation effects appear to be closely related. Briefly, in GH1, it has been proposed that glucose and xylose compete for the same binding site for stimulation since the addition of an equimolar mixture of the two monosaccharides does not increase the enzyme activity in a synergistic way (Corrêa et al. 2021). The mechanisms of xylose-only activation of β-glucosidase are unknown and require further investigation.

The effect of furfural and 5–HMF on enzyme activity was also tested (Fig. 5D). These lignocellulose pretreatment-derived compounds did not affect the enzymatic activity of AnGH3, even at concentrations up to 100 mmol L^− 1^. Additionally, the enzyme retained 100 and 70% of its activity in the presence of high concentrations (200 mmol L^− 1^) of furfural, and 5–HMF, respectively. These results surpassed those reported for β-glucosidase from *A. niger* URM 6642 (Oriente et al. 2015), commercial cellulase (Qi et al. 2018), and Lfa2 from metagenomic DNA isolated from soil samples (Alves et al. 2018). β-glucosidase from *A. niger* retained 86% of its activity in the presence of 40 mmol L^− 1^ furfural and 100% in the presence of 40 mmol L^− 1^ 5–HMF (Oriente et al. 2015). Similarly, the commercial cellulase (SunSon Group) displayed 100% of its initial activity in 5 g L^− 1^ (about 40 mmol L^− 1^) furfural (Qi et al. 2018). Lfa2 retained 70% of its activity in 10 g L^− 1^ (about 79 mmol L^− 1^) of furfural, and 100% in the presence of lower concentrations as 0.05 (about 4 mmol L^− 1^) and 0.1% (about 8 mmol L^− 1^), of 5–HMF. On the other hand, in 0.5 (about 40 mmol L^− 1^) and 1% (about 80 mmol L^− 1^), 5–HMF increased Lfa2 activity by 60 and 70%, respectively (Alves et al. 2018). Thus, although tolerance to lignocellulose-derived inhibitors like 5–HMF and furfural is crucial for the economic feasibility of enzymatic conversion of the lignocellulosic biomass, few studies test their effect on β-glucosidases.

The effect of ethanol on β-glucosidase activity is another fundamental analysis for its biotechnological application because the enzyme will be exposed to considerable ethanol concentrations, as in applications such as simultaneous saccharification, fermentation process, and winemaking (Su et al. 2022). β-glucosidase activity on *ρ*NPG was evaluated in the presence of ethanol at various concentrations (0–50%, v/v) (Fig. 5B). Surprisingly, enzyme activity was stimulated even at high concentrations of 25% ethanol. Furthermore, the enzyme maximized 1.5-fold stimulation in the presence of 10% ethanol. Literature has reported that changes in polarity in the reaction medium induced by alcohols could stabilize the conformation of the enzyme (Mateo and Di Stefano 1997; Karnaouri et al. 2013; El-Ghonemy 2021). Even at high concentrations of 50% ethanol, the enzyme retained 52% of its activity relative to the control, indicating that AnGH3 was highly ethanol tolerant. These results exceeded those reported for ethanol-tolerant β-glucosidase from *Aspergillus sp.* DHE7 (El-Ghonemy 2021), glucose tolerant GH3 β-glucosidase from *Malbranchea pulchella* (MpBgl3) (Monteiro et al. 2020), and GH3 β-glucosidases BglA, and BglJ from *A. oryzae* (Kudo et al. 2015). An improvement in the catalytic potential of some β-glucosidases in the presence of ethanol has been attributed to its glycosyl transferase activity (Wang et al. 2016; Mateo and Andreu 2020; El-Ghonemy 2021). In a reactional environment with high levels of alcohols compared to water levels, ethanol can act as an acceptor for the glycosyl moiety during catalysis of *ρ*NPG, resulting in higher reaction rates (Arévalo Villena et al. 2006; Wang et al. 2016; Mateo and Andreu 2020; El-Ghonemy 2021). However, at higher concentrations (≥ 20% v/v) of the polar solvents such as ethanol, the activity could be inhibited by conformational changes or denaturation (disruption of the secondary and tertiary structure) (Stepankova et al. 2013; Mateo 2023).

#### Kinetic parameters

Table 2 presents the kinetic parameters K_M_, V_max_, K_cat_, and catalytic efficiency of the AnGH3 using *ρ*NPG and cellobiose as substrate. The recombinant enzyme showed higher specificity to *ρ*NPG than cellobiose, exhibiting K_M_ of 0.0607 mmol L^− 1^, V_max_ of 212 U mg^− 1,^ and K_cat_ of 275 s^− 1^ under optimal conditions. The K_M_ and V_max_ of the AnGH3 using cellobiose were 2.7 mmol L^− 1^ and 57 U mg^− 1^, respectively.

**Table 2:** The kinetic parameters of AnGH3 against *ρ*NPG and cellobiose hydrolysis at pH 6.0 and 65 °C

		V_max_ (U mg^− 1^)	K_M_ (mmol L^− 1^)	K_cat_ (s^− 1^)	K_cat_/K_M_ (mmol L^− 1^ s^− 1^)
***ρ*** **NPG**	Michaelis-Menten	212	0.0607	275	4521
	Hanes-Woolf	211	0.0510	274	5371
**cellobiose**	Michaelis-Menten	56.7	2.71	73.5	27.2
	Hanes-Woolf	56	2.51	72.6	28.9

The values shown represent means ± SD from triplicate assays (*n* = 3)

Other GH3 produced in different expression systems show a wide range of kinetic parameter values (Table 3). However, AnGH3 showed a higher affinity for *ρ*NPG (K_M_ = 0.0607 mmol L^− 1^) compared to many other recombinant fungal β-glucosidases: BglA (K_M_ = 0.75 mmol L^− 1^) (Kudo et al. 2015), BglJ (K_M_ = 0.48 mmol L^− 1^) (Kudo et al. 2015), Cel3A (K_M_ = 0.4 mmol L^− 1^) (Gudmundsson et al. 2016), RmBglu3B (K_M_ = 0.17 mmol L^− 1^) (Guo et al. 2015). For cellobiose, the AnGH3 K_M_ value (2.7 mmol L^− 1^) was similar to the recombinant GH3 from *M. thermophila* (K_M_ = 2.6 mmol L^− 1^) (Karnaouri et al. 2013), rBgl3 from *A. fumigatus Z5* (K_M_ = 2.2 mmol L^− 1^) (Liu et al. 2012), and commercial preparation Novozym 188 (K_M_ = 2.4 mmol L^− 1^) (Kao et al. 2019). In general, β-glucosidases show high catalytic activity and higher K_M_ with synthetic substrates (*ρ*NPG and methyl umbelliferyl β-D-glucoside (MUG) compared to cellobiose. According to recent studies, the beta-glucosidase’s kinetics depends on its substrate’s configuration. These enzymes have a very rigid structure in the S1 substrate binding site, and one of the cellobiose glucose molecules needs to rotate to fit into the substrate binding site. This conformational change for catalysis is not required for *ρ*NPG because its small nitrophenyl group is relatively free to move in the S1 substrate binding site (Nam et al. 2010; Singhania et al. 2013; Bonfá et al. 2018).

**Table 3:** General catalytic properties of AnGH3 compared with other GH3 β-Glucosidases

Source	Expression system	Substrate	Temperature/ pH optimum	V_max_ (U mg^− 1^)	K_M_ (mmol L^− 1^)	K_cat_ (s^− 1^)	K_cat_/K_M_ (mmol L^− 1^ s^− 1^)
*Aspergillus nidulans* (This work)	*A. nidulans*	*ρ*NPG	-	212	0.0607	275	4521
		cellobiose	65–70 °C / 6.0	56.7	2.71	73.5	27.2
*Aspergillus oryzae* (BglA) (Kudo et al. 2015)	*A. oryzae*	*ρ*NPG	50 °C / 5.5	456	0.75	651	868
*A. oryzae* (BglJ) (Kudo et al. 2015)	*A. oryzae*	*ρ*NPG	40 °C / 4.5	264	0.48	373	777
*Rasamsonia emersonii* (Gudmundsson et al. 2016)	*Hypocrea jecorina*	*ρ*NPG	37 °C / 5	-	0.40	5.4	13.5
		cellobiose		-	0.78	5.5	7.05
*Termatoga petrophila* (Xie et al. 2015)	*Escherichia coli*	*ρ*NPG	90 °C / 5	109	1.6	-	-
*Rhizomucor miehei* (Guo et al. 2015)	*E. coli*	*ρ*NPG	50 °C / 5	55.9	0.17	0.071	0.42
		cellobiose	-	33.5	3.7	0.043	0.012
*Humicola insolens* (Xia et al. 2016a)	*Pichia pastoris*	*ρ*NPG	60 °C / 5.5	46.2	0.90	73.1	81.6
		cellobiose	-	-	8.44	-	11.1
*Myceliophthora thermophila* (Karnaouri et al. 2013)	*P. pastoris*	*ρ*NPG	70 °C / 5	47.9	0.39	-	-
		cellobiose	-	49.4	2.64	-	-
*Chaetomella raphigera* (Kao et al. 2019)	*P. pastoris*	*ρ*NPG	70 °C / 5	419	0.2	-	-
		cellobiose	-	312	0.96	-	-
*Aspergillus fumigatus* Z5 (Liu et al. 2012)	*P. pastoris*	*ρ*NPG	60 °C / 6	131	1.76	-	-
		cellobiose	-	53	2.2	-	-
*Talaromyces leycettanus* (Xia et al. 2016b)	*P. pastoris*	*ρ*NPG	75 °C / 4.5	1309	0.18	1664	9096
		cellobiose	-	618	10.4	786	75.8

In the present study, AnGH3 exhibited the apparent K_cat_ of 275 s^− 1^, using *ρ*NPG as the substrate. According to Cairns and Esen (Ketudat Cairns and Esen 2010), β-glucosidases usually have K_cat_ values of around 300 s^− 1^ or lower. In addition, AnGH3 showed a catalytic coefficient (K_cat_/K_M_) for *ρ*NPG of 4521 mmol L^− 1^ s^− 1^. Based on the literature values, this catalytic coefficient is one of the highest ever reported for β-glucosidase acting on this substrate (Erkanli et al. 2024), except for Bgl3A from *Talaromyces leycettanus* JCM12802 (Xia et al. 2016b). Other elevated K_cat_/K_M_ values were reported to Bgl4 of *P. funiculosum* NCL1 (K_cat_/K_M_ = 2888 mmol L^− 1^ s^− 1^) with *ρ*NPG, and (K_cat_/K_M_ = 3610 mmol L^− 1^ s^− 1^) with cellobiose at 50 °C (Ramani et al. 2015); and BglA of *A. oryzae* (K_cat_/K_M_ = 868 mmol L^− 1^ s^− 1^) using *ρ*NPG (Kudo et al. 2015).

### Enzymatic saccharification of biomass

To investigate the potential application of the recombinant AnGH3 from *A. nidulans* in biomass conversion, the enzyme was combined with commercial extract Celluclast^®^ 1.5 L using tropical forage grass (*P. maximum*) as the cellulosic material. The sample of tropical forage grass used had an estimated cellulose of 26.2 ± 0.5%, hemicellulose of 20.5 ± 0.2%, and an estimated total lignin of 26.3 ± 0.8% (Freitas et al. 2021a). After 24 h hydrolysis at 50 °C, pH 5.0, the concentration of glucose released by Celluclast^®^ 1.5 L alone was 25.6 ± 0.4 mmol L^− 1^. When the hydrolysis using Celluclast^®^ 1.5 L was combined with AnGH3, a 1.5 − fold conversion (37.1 ± 1.2 mmol L^− 1^) increase was observed (Fig. 5E). Similar result was reported to recombinant β-glucosidase from *Thermoanaerobacterium aotearoense* when combined to commercial cellulase (Cellic^®^ Ctec2) and applied in the sugarcane bagasse hydrolysis. The supplement of the purified β-glucosidase provided about 20% enhancement of the released reducing sugars (1.2-fold than of commercial cellulase alone) after a 70 h reaction at 50 °C pH 6.0 (Yang et al. 2015). Cao and coworkers (Cao et al. 2015) using β-glucosidase (Bgl6) isolated from a metagenomic library with the commercial cellulase (Celluclast^®^ 1.5 L) to hydrolyze pretreated sugarcane bagasse resulted in about 1.5 − fold more conversion (15% more conversation) than using Celluclast^®^ 1.5 L alone after 240 h at 50 °C pH 6.0. This previous study demonstrated the high potential of AnGH3 to act in lignocellulosic biomass degradation cocktails. Thus, more studies are being carried out in our research group to optimize its application in biomass hydrolysis.

## Conclusion

In this work, the gene *angh3* encoding the β-glucosidase from *A. nidulans* FGSC A4 was functionally expressed and secreted by the homologous host strain *A. nidulans* A773. The purified β-glucosidase AnGH3 showed activity and stability at pH and temperature values similar to the conditions needed for biomass hydrolysis. In addition, AnGH3 was stimulated by D-xylose and ethanol molecules, was tolerant to phenolic compounds, and showed good kinetic properties. Thus, the biochemical assays demonstrated that the enzyme could be a promising candidate for industrial applications in enzymatic cocktails.

Furthermore, we believe these properties can be further improved by immobilizing the enzyme, increasing its operational performance and, therefore, the cost-benefit ratio of its lignocellulosic hydrolysis application. The appropriate choice of support, functional groups, and the strategy and protocol involved in immobilization can increase AnGH3 stability even more (more comprehensive pH, activity, temperature profiles, increased reuse cycles, etc.). In addition, the interaction of the enzyme with the support can cause conformational changes that can promote some positive effects on the enzyme’s characteristics. For example, the immobilization of AnGH3 in the presence of its stimulating compounds, ethanol and xylose, can make it immobilized in the “hyperactivated” state or, in some instances, prevent inhibitions. That way, the potential of AnGH3 immobilization opens excellent possibilities to expand its peculiar characteristics (Di Cosimo et al. 2013; Sheldon and van Pelt 2013; Rodrigues et al. 2021; Bolivar et al. 2022).

### Electronic supplementary material

Below is the link to the electronic supplementary material.

Supplementary Material 1: Amino acid sequence of the β-glucosidase AnGH3 from *Aspergillus nidulans* FGSC A4. The peptides corresponding to those identified by mass spectrometry are underlined and in bold

Supplementary Material 2: Scheme of the different compounds used to measure the AnGH3 activity from *A. nidulans*. This manuscript also supports the “supplementary file” with a full uncropped Gel image

Supplementary Material 3

## Data Availability

The datasets used and/or analyzed during the current study are available from the corresponding author upon reasonable request.
