# Supplementary figures and images for: Recombinant GH3 β-glucosidase stimulated by xylose and tolerant to furfural and 5-hydroxymethylfurfural obtained from Aspergillus nidulans
Source: Bioresour Bioprocess. 2024 Jul 29;11(1):77. doi: 10.1186/s40643-024-00784-2 (PMC11286919; doi:10.1186/s40643-024-00784-2)

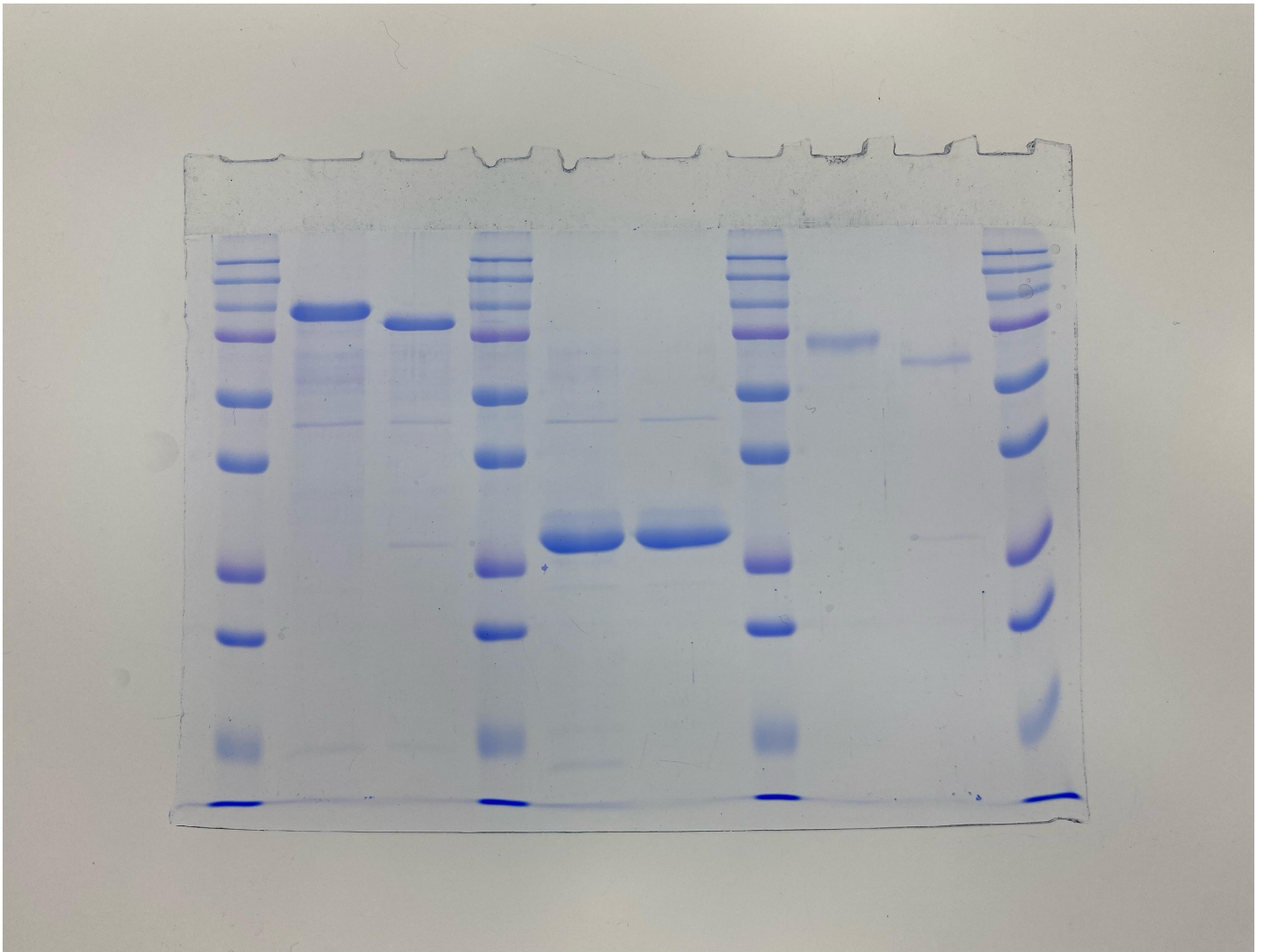

Supplement: Supplementary file 1 — Supplementary Material 1: Amino acid sequence of the β-glucosidase AnGH3 from Aspergillus nidulans FGSC A4. The peptides corresponding to those identified by mass spectrometry are underlined and in bold [file 40643_2024_784_MOESM1_ESM.pdf]

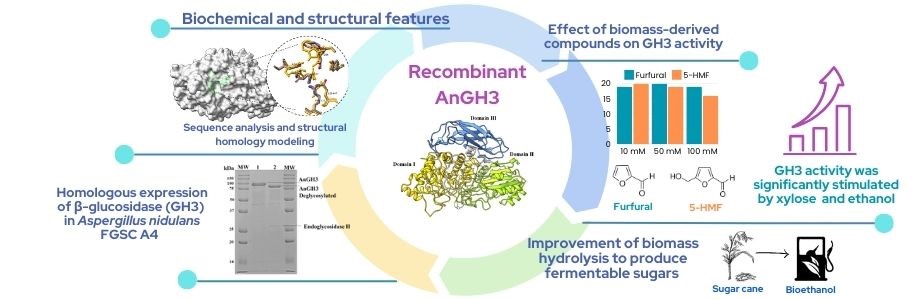

Supplement: Supplementary file 2 — Supplementary Material 2: Scheme of the different compounds used to measure the AnGH3 activity from A. nidulans. This manuscript also supports the “supplementary file” with a full uncropped Gel image [file 40643_2024_784_MOESM2_ESM.jpeg]
