# Supplementary material for: Recombinant GH3 β-glucosidase stimulated by xylose and tolerant to furfural and 5-hydroxymethylfurfural obtained from Aspergillus nidulans
Source: Bioresour Bioprocess. 2024 Jul 29;11(1):77. doi: 10.1186/s40643-024-00784-2 (PMC11286919; doi:10.1186/s40643-024-00784-2)
Supplement: Supplementary file 3 — Supplementary Material 3 [file 40643_2024_784_MOESM3_ESM.pdf]

Supplementary data

Recombinant GH3 β-glucosidase stimulated by xylose and tolerant to furfural and 5-hydroxymethyl furfural obtained from *Aspergillus nidulans*

Diandra de Andrades[1+], Robson C. Alnoch[1,2+], Gabriela S. Alves[3], Jose C. S. Salgado[1,4], Paula Z. Almeida[2], Gabriela Leila Berto[5], Fernando Segato[5], Richard J. Ward[4], Marcos S. Buckeridge[6] and Mariade Lourdes T. M. Polizeli[1,2*]

Supplementary Figure S1. Amino acid sequence of the β-glucosidase AnGH3 from *Aspergillus nidulans* FGSC A4. The peptides corresponding to those identified by mass spectrometry are underlined and in bold.

>gi|67524740|ref|XM_655340.1| ***Aspergillus nidulans*** FGSC A4 hypothetical protein AN2828.2 partial mRNA

MRSLIRSGALNAFLAASLATGQVLTWDEAYTKATSDLSLLSQEEKVGIVTGVTWQGG
PCVGNTYEPTSIPYPSLCLQDGPLSVRFANPVTVFPAGINAGATWDRELIRA**RGVAMG**
AESRGLGVHVQLGPVAGALGKIPSAGRNWEGFSNDPYLAGIAMAEAIQGMQSSGVQAC
AKHYLLNEQEYNRDTISSNADD**RTIHELYLWPFYDAVK**ANVASVMCSYNKINGTWACE
HDALLNGLLKGELGFKGHVLSDWNAQHSTVQSANTGLDMTMPGSDFSTPPGSIYWGDN
LAAAIADGSVPQERLDDMVTRILAAWYLVGQDQGHPPVAFSSWDGGAASVNVTTPEHG
ELARTIARDSIVLL**KNTNGSLPLAKPASLAVIGSDAIVNPDGANACADR**GCNKGTLAQ
GWGSGTAEFPYLVAPLDAIEEKLAGAGTAIITSTTDDATSGAEAAAAAETAIVFITSD
SGEGYITVEGHEGDRNNLDPWHNGNLLVQAVARTNTPTIVVLHSVGPVTLETILAEPN
VVAVVWAGLPGQESGHALTDVLFGDYAPSGKLPFTIGKSEEDYGADWTTSQVDDFAEG
LFIDYRHFDQYGIEPRYEFGFGLSYTSFNYSTLSTSISTTPGPTTGETIVGGPSDLFA
PIGTVSAYVANTGHVAGAEVVQLYIGYPDSAPSIPPKQL**RGFDKLHLVPGESGIATFE**
LTRRDISYWDVGLQKWVVASGTFEVFVGASSRDIRLTGSFTV

*Signal peptide

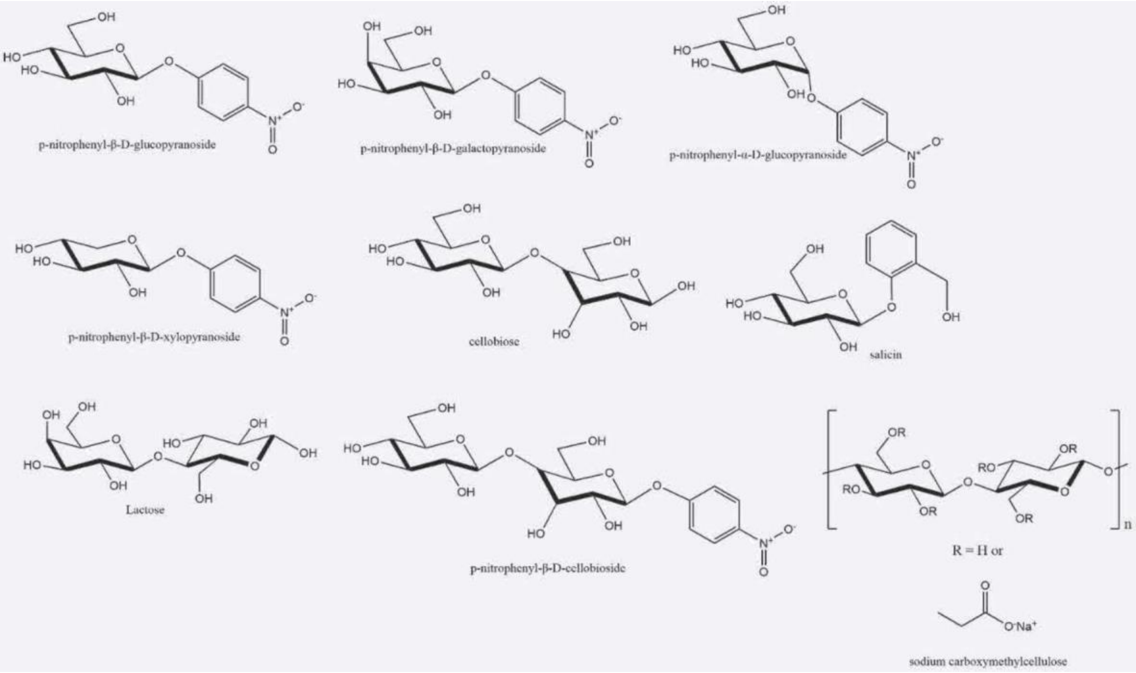

Supplementary Figure S2. Scheme of the different compounds used to measure the AnGH3 activity from *A. nidulans*.
